# Hepatic Mesenchymal Hamartoma With Elevated Alpha-Fetoprotein: A Diagnostic Dilemma

**DOI:** 10.7759/cureus.74791

**Published:** 2024-11-29

**Authors:** Somesh Venkataraman, Nadeem Ur Rahman, Jitendra Sharma, Abhinav Bhagat, Aparna Valsan

**Affiliations:** 1 Radiology, All India Institute of Medical Sciences, Bhopal, Bhopal, IND; 2 Radiodiagnosis, All India Institute of Medical Sciences, Bhopal, Bhopal, IND; 3 Pathology and Lab Medicine, All India Institute of Medical Sciences, Bhopal, Bhopal, IND

**Keywords:** alpha-fetoprotein, computed tomography abdomen, hepatic mesenchymal hamartoma, hepatic tumor, hepatoblastoma

## Abstract

Hepatic mesenchymal hamartoma (HMH) is an uncommon, benign liver tumor predominantly affecting children under three years of age. It is characterized histologically by disorganized mesenchymal stroma, abnormal bile ducts, blood vessels, and hepatocytes. HMH can present as a large cystic mass, a solid mass, or a combination of both. Hepatoblastoma, the most common malignant liver tumor in children, remains a primary differential diagnosis. This case report highlights the diagnostic challenge presented by HMH, particularly when alpha-fetoprotein levels are mildly elevated. A two-year-old male presented with abdominal distension and a palpable mass, with initial ultrasound imaging revealing a large cystic and solid liver lesion. Contrast-enhanced computed tomography showed a well-defined mass with both cystic and solid components arising from the liver with a large exophytic soft tissue component extending and occupying the majority of the abdominal cavity, raising the possibility of HMH and hepatoblastoma. Histological examination confirmed HMH, with no malignancy identified. This case emphasizes the crucial role of imaging and histopathology in differentiating HMH from other hepatic lesions. Although HMH generally has an excellent prognosis with complete surgical resection, it may rarely undergo malignant transformation, necessitating careful diagnostic evaluation and management.

## Introduction

Hepatic mesenchymal hamartoma (HMH) is an uncommon, benign tumor predominantly found in pediatric populations, with a higher incidence in children under the age of three years [1]. It is a histologically benign entity characterized by mesenchymal stroma and abnormal components of bile ducts, blood vessels, and hepatocytes [2]. HMH can manifest as a large, benign cystic mass, a solid mass, or a mass containing both cystic and solid components [3]. Hepatoblastoma is the most common primary malignant liver tumor in children and should be considered a critical differential diagnosis when evaluating pediatric hepatic neoplasms [4]. The preferred treatment approach for HMH is complete surgical resection, with an excellent prognosis. Hence, it is crucial to differentiate HMH from hepatoblastoma [1]. This case emphasizes the critical role of imaging features from ultrasound and computed tomography and the importance of histological analysis in pediatric hepatic tumors with mildly elevated alpha-fetoprotein (AFP) for establishing an accurate diagnosis.

## Case presentation

A two-year-old male child presented at the department of pediatric surgery with a four-month history of progressive abdominal distension with no history of fever, anorexia, weight loss, or jaundice. The child was otherwise normal, with an uneventful birth and perinatal history. The abdomen was distended on examination with a large right upper abdominal mass, non-tender, and firm to palpation.

Blood investigations revealed elevated AFP at 306 ng/ml (reference range < 8.78 ng/ml; chemiluminescent microparticle immunoassay, Abbott ARCHITECT i2000SR, Chicago, IL) and mildly elevated gamma-glutamyl transferase at 111.36 U/L (reference range < 55 U/L). Aspartate aminotransferase (AST), alanine aminotransferase (ALT), and alkaline phosphatase (ALP) were within normal limits.

**Figure 1:**
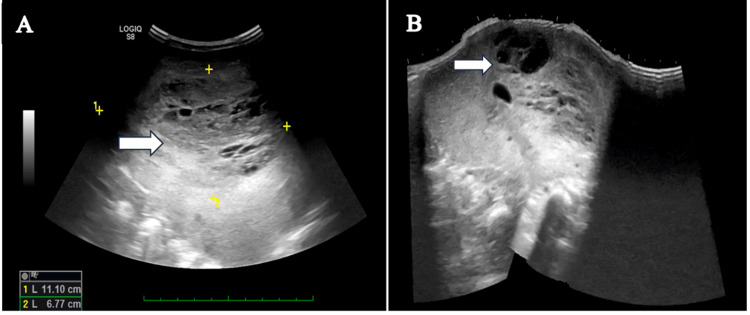
Ultrasound image showing (A) a large multilocular solid cystic lesion (white arrow) almost completely replacing liver parenchyma and (B) multiple internal cystic areas (white arrow) giving Swiss-cheese appearance.

Abdominal ultrasound showed a grossly enlarged liver with a large multilocular solid cystic lesion almost completely replacing liver parenchyma measuring ~11.1 x 6.7 cm, showing minimally raised vascularity on color Doppler flow imaging. Possible differentials were mesenchymal hamartoma, hemangioendothelioma, and hepatoblastoma (Figure 1).

**Figure 2:**
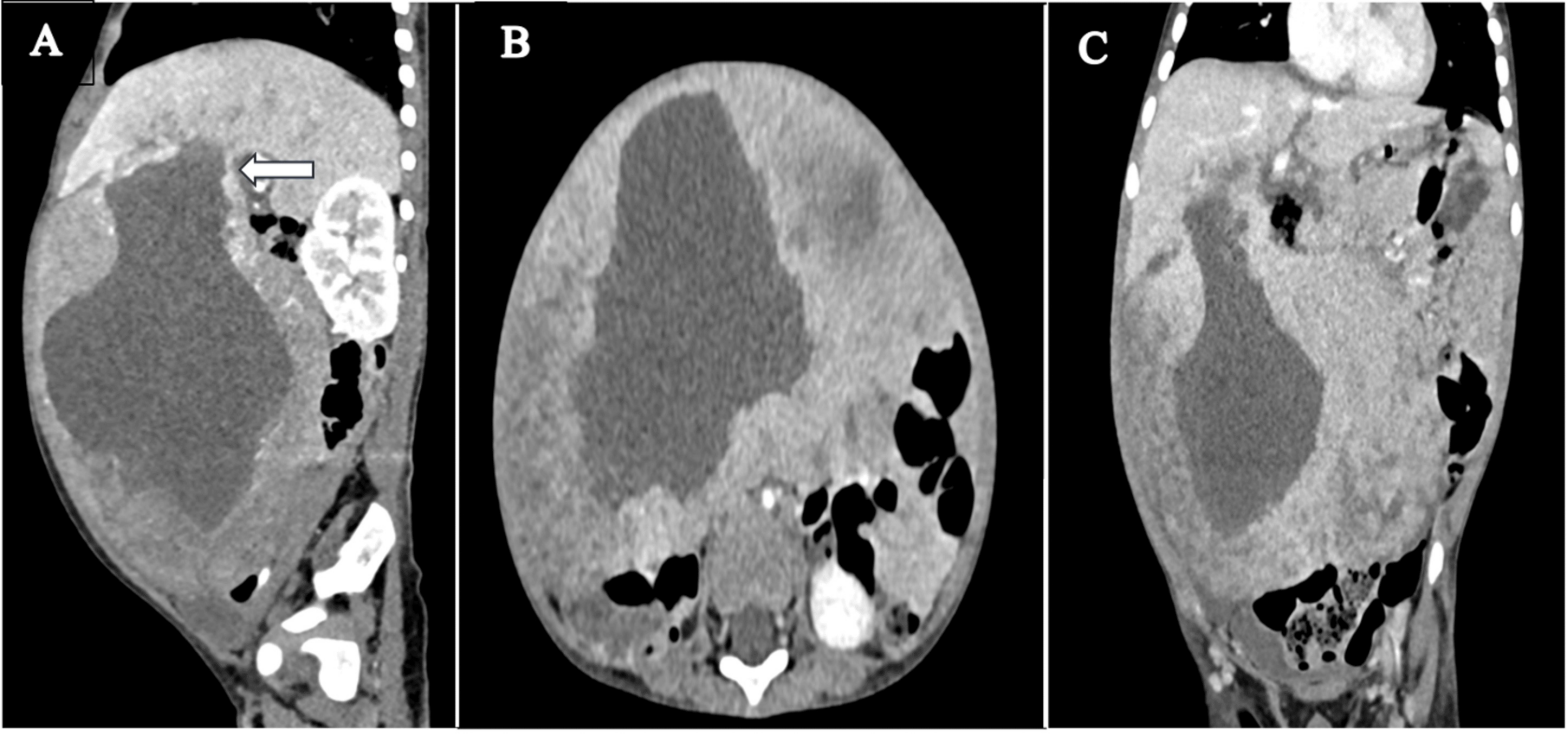
Contrast-enhanced computed tomography images depicting a large heterogenous mixed solid-cystic mass (cystic predominant) arising from the liver and protruding into the abdominal cavity. (A) Sagittal image showing exophytic solid cystic mass arising from the inferior aspect of the right lobe of the liver (white arrow). (B) Axial image showing mass causing mass effect with the displacement of the bowel loops and abutting anterior abdominal wall muscles. (C) Coronal image showing mass with enhancing solid component and interspersed non-enhancing cystic component.

The child was further evaluated with contrast-enhanced computed tomography (CECT) of the abdomen. A large, well-defined solid mass with a large cystic component was noted in the abdominopelvic cavity, predominantly on the right side, measuring ~8.0 x 14 x 15 cm. The lesion abutted the inferior surface of the right lobe of the liver on its superior aspect with indistinct soft tissue planes with the liver with thinned-out compressed hepatic parenchymal tissue seen along the edge of the mass, representing a claw sign. The lesion was seen supplied by branches of the right and left hepatic arteries. These features suggested the possible hepatic origin of the lesion. No evidence of calcifications or fat/blood attenuating density was seen. In the porto-venous phase, the peripheral part showed homogenous enhancement similar to liver parenchyma. There was an enhancing solid component with the interspersed non-enhancing cystic component, giving a Swiss-cheese appearance at the inferior aspect of the lesion. The lesion exerted mass effect with abutment and displacement of the adjacent structures with no evidence of any invasion or infiltration. No significant intra-abdominal lymphadenopathy was noted. Hence, based on CT imaging, a possible differential of pediatric hepatic lesions such as hepatic mesenchymal hamartoma and other less likely possibilities of hepatoblastoma were considered in view of mildly raised AFP levels (Figure 2).

**Figure 3:**
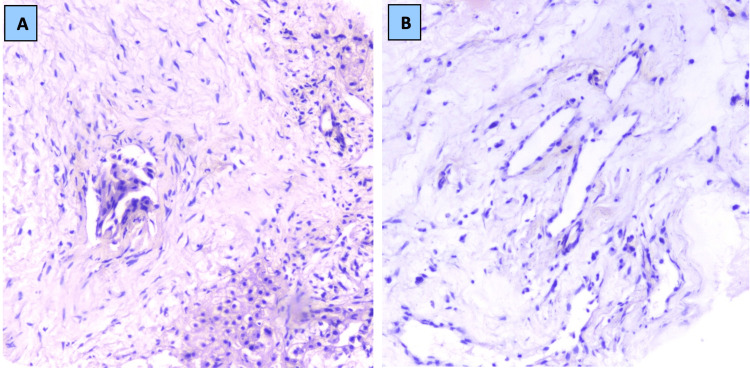
Photomicrograph of the lesion showing (A) loose myxoid stroma with irregular bile ducts (H&E, 40x) and (B) myxoid stroma with dilated cystic spaces lined by flattened epithelium (H&E, 40x). H&E: hematoxylin and eosin.

The patient then underwent an ultrasound-guided tru-cut biopsy for histopathological evaluation. Microscopic examination revealed linear cores of liver parenchyma with the presence of a disorganized arrangement of benign mesenchymal components comprising abundant loose myxoid stroma with scattered edematous fibrous tissue. Within the stroma were irregularly shaped bile ducts, micronodules of hepatocytes, and dilated cystic spaces lined by flattened epithelium. The surrounding liver parenchyma appeared unremarkable. There was no evidence of atypia, significant mitotic activity, or malignancy. Immunochemistry revealed vimentin positive in stromal cells. These pictures were in keeping with a histopathological diagnosis of hepatic mesenchymal hamartoma (Figure 3). The mass was excised, and the child is doing well.

## Discussion

Mesenchymal hamartomas were first described in the literature by Edmondson in 1956 [5]. HMH is an uncommon, benign tumor predominantly found in pediatric populations, with a higher incidence in children under the age of three years and a slight predominance in boys [1]. Mesenchymal hamartoma arises from the disorganized proliferation of primitive mesenchymal tissue in the periportal region. Pathogenesis of mesenchymal hamartoma is explained by four hypotheses: developmental, vascular, toxic insult, and neoplasia. The postulated hypotheses are (a) mesenchymal hamartoma of the liver develops from the ductal plates of the prenatal liver [6]; (b) mesenchymal hamartoma is a lesion with an abnormal vascular supply that can develop its characteristic pattern of stromal cysts due to early ischemic changes [7]; (c) the presence of desmin and alpha-actin in the lesions indicates that fat-storing (Ito) cells in the immature liver may play a role in the development of mesenchymal hamartoma, and Ito cells are activated by toxic insult [6]; (d) cytogenetics and flow cytometry have revealed balanced translocations at 19q13.4 and aneuploidy that suggest a neoplastic process [8].

On gross pathology, mesenchymal hamartomas of the liver typically appear well-defined with irregular margins formed by compressed liver tissue, bile ducts, and blood vessels, but they lack a true capsule. Internally, the tumor is characterized by multiple cysts containing clear or pale yellow serous or mucoid fluid. The composition of this fluid is similar to plasma but with lower levels of protein, cholesterol, and glucose. These cysts are not connected to the biliary system. Instead, they are separated by fibrous septa and surrounded by loose mesenchymal tissue containing tortuous biliary ducts, blood vessels, and liver tissue [1,9,10].

Microscopically, these tumors display both mesenchymal and epithelial characteristics. The myxoid stroma contains a mix of cystic spaces, some lined with epithelium and others without. Smaller cysts are generally lined with cuboidal biliary-type epithelium, whereas larger cysts, often lacking epithelial lining, likely result from fluid accumulation in degenerated mesenchyme. The stroma consists of fibroblasts, collagen, bile ductules, blood and lymphatic vessels, and clusters of hepatocytes. The mesenchymal component of the tumor may exhibit immunoreactivity to markers such as vimentin, smooth muscle actin, α-1-antitrypsin, and desmin [1,10-12].

Mesenchymal hamartoma of the liver presents with symptoms like abdominal distension and/or a mass in the upper abdomen. Other symptoms can include abdominal pain, loss of appetite, vomiting, and inadequate weight gain. Usually, on examination, a large, firm, smooth liver tumor that is not tender to palpation is seen. There might be engorged veins on the abdominal wall, and although less common, there could also be swelling in the lower limbs due to compression of the inferior vena cava. Other presentations are rare and can occur in prenatal cases, newborns, older children, and adults. Prenatally, it is detected by antenatal ultrasound, often in the third trimester, and associated with polyhydramnios [13]. In newborns, it can cause life-threatening abdominal distension with respiratory distress/apnea and high-output cardiac failure [1]. In older children, it can rarely present with obstructive jaundice, disseminated intravascular coagulation, and constipation [1]. In adults, it presents with abdominal pain and distension. Mesenchymal hamartomas are generally not linked with other congenital anomalies. Still, they have been associated with conditions such as congenital heart disease, endocardial fibroelastosis, intestinal malrotation, esophageal atresia, biliary atresia, exomphalos, myelomeningocele, and Beckwith-Wiedemann syndrome [1].

Biochemical liver function tests are usually normal but gamma-glutamyl transferase (GGT) may be mildly elevated. AFP levels are usually normal or mildly elevated. The quantity of hepatocytes and bile duct epithelium in the mesenchymal hamartoma is positively related to the serum AFP level [14].

Ultrasound, CT, and MRI reveal a multiloculated cystic tumor with varying amounts of solid tissue [15,16]. On ultrasound imaging, the cystic regions are characterized by the presence of thin or thick echogenic septations and internal debris, while the solid components demonstrate echogenicity. Color Doppler flow imaging (CDFI) reveals minimal vascularity within the septations and solid regions. In certain instances, the cysts may be very small, causing the hamartoma to appear as a solid echogenic mass. Outside the outer rim of the compressed liver, the hepatic architecture appears normal [15,16].

CT confirms the sonographic findings and provides the lesion's extent with its relation to other intra-abdominal organs. Typically, the mass appears as a well-defined solitary lesion of hepatic origin, which may be entirely intrahepatic, partially extrahepatic, or exhibit an exophytic or pedunculated growth pattern. The cystic contents exhibit density isodense to water, while the stromal components appear hypodense relative to the liver. The enhancing soft tissue elements typically surround a central region of simple fluid attenuation, accompanied by varying levels of internal septation and loculation. This multi-cystic configuration is frequently described as resembling a “Swiss cheese” pattern [15].

On MRI, the appearance of the tumor is influenced by the proportion of cystic and stromal components and the protein concentration within the cysts. On T2-weighted images, the cystic regions exhibit hyperintensity, while their signal intensity on T1-weighted images is variable. Conversely, the solid regions appear hypointense on both T1- and T2-weighted sequences due to the presence of fibrosis. Following gadolinium administration, enhancement is typically mild and localized to the stromal and septal elements [16].

Mesenchymal hamartoma of the liver has traditionally been considered a benign tumor with no risk of malignancy; however, there is an association between mesenchymal hamartoma and undifferentiated embryonal sarcoma of the liver [17,18].

In pediatric patients, hepatic lesions can have various differential diagnoses. Hepatoblastoma is commonly characterized by markedly elevated serum AFP levels, a solid appearance, and calcifications. Nonetheless, there are rare instances where hepatoblastoma may present with low serum AFP or only mildly elevated levels, and HMH might also exhibit a predominantly solid appearance. Focal infantile hemangioendothelioma with stromal myxoid alterations can be distinguished from hypovascular mesenchymal hamartoma by identifying enlarged vessels, peripheral nodular enhancement with centripetal fill-in, and calcifications. Undifferentiated embryonal carcinoma, which typically manifests in older children aged six to 10 years, is characterized by malignant stroma along with frequent hemorrhage and necrosis [16].

Hepatic mesenchymal hamartomas are best treated by complete excision. Other surgical options are enucleation and marsupialization of cysts. Liver transplantation can be considered for unresectable tumors [19].

## Conclusions

HMH is an uncommon liver tumor with a variable imaging presentation and typically has a favorable prognosis if accurately identified and managed. Nonetheless, in exceptional cases, it may undergo malignant transformation to undifferentiated embryonal sarcoma, which can exhibit a similar imaging profile. HMH can closely resemble various benign and malignant lesions, posing considerable diagnostic challenges. A mild to moderate elevation in AFP levels associated with a hepatic mass in a child is not sufficient to conclusively diagnose hepatoblastoma. Radiological imaging is essential for suggesting the diagnosis and assisting in surgical planning, while histological analysis is crucial for definitive diagnosis.
